# Limitations of workers diagnosed with cancer evaluated with *Work Role Functioning Questionnaire*-Br

**DOI:** 10.3332/ecancer.2017.769

**Published:** 2017-09-18

**Authors:** Cristiane H Gallasch, Neusa MC Alexandre, Sergio CB Esteves, Helena F Gomes, Elaine CL da Rocha, Patricia CP Baptista

**Affiliations:** 1 Faculty of Nursing of the Rio de Janeiro State University, Blv September 28, 157, 7th floor, Vila Isabel, Rio de Janeiro, Brazil; 2 Study Group on Occupational Health and Nursing Personnel (GESTES), School of Nursing, Av. Dr. Enéas de Carvalho Aguiar, 419, Cerqueira César, São Paulo, Brazil; 3 Study and Research Group for Occupational Health and Ergonomics, Campinas, São Paulo, Brazil; 4 Faculty of Nursing of the State University of Campinas. City University Zeferino Vaz, s / n. Campinas, São Paulo, Brazil; 5 Radiotherapy Section, Women’s Hospital Professor Dr. José Aristodemo Pinotti - CAISM, State University of Campinas, City University Zeferino Vaz, s / n. Campinas, São Paulo, Brazil; 6 Military Police Central Hospital in Rio de Janeiro, R. Estácio de Sá, 20, Estácio, Rio de Janeiro, Brazil; 7 School of Nursing of the University of São Paulo, Av. Dr. Enéas de Carvalho Aguiar, 419, Cerqueira César, São Paulo, São Paulo, Brazil

**Keywords:** : cancer survival, occupational health, return to work

## Abstract

The main subject of the current study is to look into the limitations found in individuals diagnosed with cancer, considered a public health problem, especially in developing countries where the impact of this disease is expected to account for 80% of 20 million new cases estimated worldwide by 2025. For some patients undergoing treatment, continuing to be professionally active is challenging especially considering that work fosters a purpose in life, a sense of contribution, distraction, and self-esteem, which aids in physical, cognitive, emotional, and interpersonal recovery.

Within this context, the aim is to describe the limitations found in individuals with cancer, who have maintained their work activities in private service during radiotherapy treatment, through a descriptive study and a quantitative approach. The final sample of 51 participants was defined by non-probability convenience sampling, based on information from all patients admitted in that period, with a research protocol approved by the Research Ethics Committee.

The assessment using the Brazilian version of the *Work Role Functioning Questionnaire* showed higher average work functioning indexes for social demand, with an average score of 94.33 (11.47). In turn, the worst indexes were registered in physical demand. No significant differences were observed between groups of treatment protocols in terms of WRFQ-Br scores. The work functioning assessment of workers diagnosed with cancer in radiotherapy using the WRFQ-Br showed higher scores for social demands and lower for physical demands. The preserved social performance may be linked to individual work functioning within the family, at work and, consequently, within society, given that the study included patients who maintained their work activities during the proposed treatment period, highlighting the social role of work for patients with cancer.

## Introduction

The main subject of the current study is to look into the limitations found in individuals diagnosed with cancer who maintained their work activities during radiotherapy treatment.

According to World Health Organisation, 11 million new cases of cancer are reported annually, with an estimated increase to 27 million by 2030, 17 million deaths and 75 million survivors worldwide [1]. The increase in the number of new cases is directly related to the increase in life expectancy of the population and exposure to risk factors [2].

Cancer is a public health problem, especially in developing countries, where more than 20 million new cases are expected by 2025. The estimates for Brazil from 2016 to 2017 point to around 600,000 new cases [3].

Surviving cancer must be an important consideration throughout treatment, given that due to the increase in its incidence, it is necessary to prevent the challenges that arise when living with a cancer diagnosis after the primary treatment cycle [4]. There is a need, therefore, for the patient and the health team to jointly develop a support plan, based on medical guidelines for a specific diagnosis as well as on the individual needs and preferences of each survivor, helping the individual feel more in control and ready to make the transition back to their daily life by promoting better physical and emotional health [5].

Cancer survival begins when the diagnosis is received and continues during the treatment in order to reduce the risk of relapse or to manage chronic disease. Nevertheless, regardless of its definition, survival is unique to each individual and everyone must find their own way to experience the changes and challenges that occur as a result of living with cancer [6].

Side effects from treatment, however, can lead to functional, physical, and/or psychological impairment, which hinder a return to work and lead to long-term leave. It is believed that it is possible to change this reality through intervention by health professionals and a return-to-work plan that can be put into motion during and after treatment. For this to happen, it becomes necessary to help this population recognise their needs and the side effects and difficulties in continuing or returning to work [7]. Continuing to work is important for remuneration, social support potential, and gives a purpose to life, a sense of contribution, distraction, and increases self-esteem, which helps in physical, cognitive, emotional, and interpersonal recovery.

Despite the growing concern with continuing and returning to daily life activities, most of the available and validated tools to assess this population are geared towards general aspects of quality of life, and there are no Brazilian studies related to functional skills or limitations related to cancer and work [9].

The use of validated instruments has been considered essential for the reliability of data obtained from individuals with physical difficulties or psychological/social changes, and helps to plan preventive actions for these disorders, as well as rehabilitation strategies and programs in the area of workers’ health.

Recognising that residual symptoms of the disease after treatment with chemotherapy and/or radiotherapy are factors that have a direct impact on the return or on the adaptation to perform work activities [10], this study aims to describe the limitations found in individuals with cancer, who have maintained their work activities in private service during radiotherapy treatment.

## Method

This is a descriptive, quantitative approach study carried out in a private radiotherapy service in the interior of the state of São Paulo between the years 2012 and 2013. It is a specialised institution, with outpatient care offered by a multiprofessional team in radiotherapy in the modalities of low-dose rate teletherapy and brachytherapy, which receives patients by spontaneous demand or by medical referral, in private consultations, through the supplementary healthcare system or under the unified healthcare system at the city level.

The population of this study was made up of patients undergoing radiotherapy, with any type of malignant tumour. The final sample, however, was comprised of 51 participants. The sample was defined by non-probability convenience sampling, based on the information of all patients admitted during the period, considering that the removal of work activities is a common recommendation for patients with an oncological diagnosis, in compliance with the following inclusion criteria: diagnosis of neoplasm confirmed by anatomopathological examination, aged 18 years or older and less than 75 years old by inclusion in the workforce, undergoing radiotherapy protocol in the teletherapy modality with exposure to a minimum dose of 2000cGy, when the onset of the first unwanted acute effects are expected arising from cumulative exposure to ionising radiation, termination of radiotherapy treatment for a maximum of two years (24 months), at which time complaints related to the exposure to ionising radiation become less likely [11], maintenance of work activities, formal or informal, with a minimum weekly load of 20 hours. The exclusion criteria considered were illiteracy, due to the impossibility of filling out forms, and the undertaking of an exclusive radiotherapy protocol in the brachytherapy modality.

All patients were initially contacted either in person or by phone by the main researcher, and the goals of the study were laid out and later, survey forms were sent to them. After the acceptance of the participant, the terms of consent and materials were sent by mail, with prepaid forms and envelopes to be returned by the participants. For social, demographic, clinical and occupational characterisation, a participant characterisation form was used, containing information about age, sex, job title, type, and time on the job, weekly work shift pattern, work leave during treatment, time to return back to work, diagnosis and work functioning level, or *performance status, – Karnofsky scale* (KPS), the latter being data from the clinical assessment.

In order to assess the limitations, the *Work Role Functioning Questionnaire*-Br (WRFQ-Br), adapted transculturally for the Brazilian population [12], was used and validated for a population with a diagnosis of cancer in radiotherapy [13].

It is worth noting that WFRQ-Br, in its original version (WRFQ), has already pointed out significant limitations in work for individuals with various types of brain cancer due to fatigue, depression, anxiety or cognitive impairment, and the same signs and symptoms, except for cognitive changes, in situations of thoracic cancer [14, 15].

The aforementioned instrument enables assessments in the fields of *physical requirements*, measuring the ability to perform work activities that include physical, dynamic and static loads, such as heavy objects, movements, resistances, coordination and flexibility; *mental*, considering cognitive demands related to attention and concentration; *social*, referring to interactions of the individual with coworkers and company clients; in *work planning*, checking the worker’s difficulties in planning and managing their daily occupational activities, from the beginning to the end of the workday; and demand for *production*, covering productivity, quality of service and job satisfaction; this set of information is not found in another single instrument of assessment in the field of workers’ health [12, 13].

In order to be able to assess the five sub-scales, or demands, the respondent must not fill in the ‘Does not apply to my job’ option for more than 20% of the questions of each demand. After this verification, the values of the valid items for the work of each individual of each subscale is summed separately and the final value is divided by the number of items, which results in a value between four and zero, then multiplied by 25, which results in a value between 0% and 100%. This value indicates the preserved work functioning index of the individual according to the functions that he/she carries out on the job for each demand assessed. For the general assessment of the work functioning index, the arithmetic average of the five demands is calculated. Values equal to 0% indicate that the work functioning index is completely impaired, while values equal to 100% reflect a fully preserved capacity or work functioning [16].

The data were collected in 2012 and 2013, based on the submission of forms to patients included in the inclusion criteria. All material submitted was returned.

Regarding the data analysis, a descriptive analysis was performed to characterise the participants. The data collected were entered into a *Microsoft Excel database* and was analysed using absolute (*n*) and relative (%) frequencies for the categorical variables, with the aid of the institution’s statistical service.

The research protocol was approved by the Research Ethics Committee under protocol no. 104/2009. It should be noted that in order to comply with Resolution 466/2012, the confidentiality and anonymity of the surveyed participants was ensured, as well as the Free and Informed Consent Form (Termo de Consentimento Livre e Esclarecido – TCLE), which explained the purpose of the research, the risks, benefits and rights of those who participated in the survey and the duties of the researchers. The TCLEs, signed in two copies, were given to the interviewee and the researchers. Reports of death or a turn to the worse in health status were reported immediately to the clinical staff responsible for these patients. Requests for the scheduling of consultations were forwarded to the service desk.

## Results

Table 1 shows the characteristics of the participants of the study as well as the diagnoses and treatment regimes to which these individuals were submitted.

Fifty-one patients, with a medical diagnosis of malignant neoplasia, underwent radiotherapy, aged between 30 and 72 years (53.35 ± 10.08), composed of 23 men (45.10%) and 28 women (54.90%).

With regard to schooling, there was a predominance of participants with higher education (54.90%). The average weekly working day was 41.45 (10.04) hours. The patients presented an average of 20.31 (11.92) years of work in the stated job, with a predominance of mixed labour, followed by non-manual and manual labour.

Prostate (23.53%) and breast (45.10%) treatments were the most prevalent. On average, patients received 5664cGy (117.1) of radiotherapy dose, teletherapy protocol, until the moment of participation in the study, which was, on average, 288.63 (143.45) days after the last application. There is a high standard deviation value due to the possibility of approaching patients during treatment (doses close to 2000cGy) and up to two years later (doses up to 7600cGy).

Most of the radiotherapy protocols of the study were complemented by surgery and chemotherapy (33.33%) and surgery (27.45%). The *Karnofsky scale* showed an average of 94.31 (8.06), with results of 80 (19.61%), 90 (17.65%) and 100 (62.75%).

The score values obtained from the assessment using the WRFQ-Br are shown in Table 2, and the values of the work functioning assessment at work compared to the treatment protocols of the patient group are shown in Table 3.

There was an observation of higher average work functioning indexes for social demand, with 94.33 (11.47). In turn, the worst indexes were registered in physical demand. No significant differences were observed between groups of treatment protocols in terms of WRFQ-Br scores.

## Discussion

Work has always been understood as part of a world experienced by the person, his/her body and his/her social relations, representing a source of pleasure or suffering that results in different expressions of productivity and creativity. It is through it that man finds his space within the community [17, 18], meaning the collective. The return to work of cancer survivors has been reported as problematic due to changes in physical capacity, fatigue, lack of support and adaptations in the occupational environment, as well as work overload [19].

The study included 51 patients with neoplasms who were undergoing radiotherapy and who worked mostly in mixed activities (41.18%), characterised by intermittent handling of heavy or continuous low-weight loads, and non-continuous permanence in a static position [19]. The same was observed in a previous instrument validation study [20].

Due to high physical and psychological demands at work, in addition to long work hours, a large amount of work leave was observed during cancer treatment. There are no statistics regarding the amount of cancer patients on leave from work; however, the study showed 13.45% of cases with leave, even after the end of the proposed protocol.

In addition to a physical and psychological overload, the clinical team usually offers the possibility of work leave to the patient so that there is more time dedicated to treatment and care in terms of possible complications, since in Brazil all individuals who perform their jobs according to the rules of Labour Law Consolidation (CLT) remain from 40 to 44 hours a week in work-related activities [21]. This was the average trend followed by the group of patients (41.45 hours per week).

Data from the National Social Security Institute (Instituto Nacional de Seguridade Social – INSS) shows that 149,173 active disability benefits were granted in 2011 for CID 10, referring to cancer, representing an annual cost of R$ 140,140,688.45. This group is offered a sickness allowance, granted to every worker who is unable to work for 15 days or more, with medical proof [22]. There is no clear data on how to assess the capacity of these individuals or the attempts to adapt to work, reducing overload or working hours, as ways to avoid total withdrawal from work activities.

In Brazil, as in other regions around the world, the elevation of survival rates and also cure after treatment in individuals of economically active age increase the chances of permanence or return to work. This rate of return varies according to the characteristics of each country’s support systems, including health, rehabilitation, and social systems [23].

The study found that most radiotherapy treatment regimes were complemented by surgery and chemotherapy or surgery alone. Oncologic surgery is still the primary mode of treatment for malignant neoplasms and is considered vital for reducing premature cancer mortality, with prevention, diagnosis, treatment, palliative and reconstructive treatment functions, however, not always representing a disabling aspect in terms of health. Over time, it has undergone technical modifications and intensive care that offer greater security. In addition, with greater and better knowledge of the history of the disease and its prognosis, surgical intervention has become less radical and invasive [24, 25].

Although chemotherapy affects cell metabolism, not always with specificity, reaching bone marrow and other germ cells, with undesirable effects up to 14 days after administration – such as leukopenia, thrombocytopenia, anaemia, alopecia, stomatitis, diarrhoea and oligospermia [24], the worsening of the health of the patient group related to the administration of these drugs was not a criterion for altering the assessment scores, due to the time elapsed from the end of the treatment to the data collection, which was on average 288.63 (143, 45) days.

The high index on the *Karnofsky scale* shows that the individual may have preserved physical ability for other activities such as work. Studies indicate that this index has already been related as predictive of post-treatment survival [26], which is directly related to the planning of actions aimed at cancer survival.

In terms of the results of work functioning assessment at work, the group presented higher scores for social demand, but the worst work functioning was observed for physical demand, which can be explained by functional changes arising from previous surgical interventions, or by limitations resulting from the specific clinical scenario.

The detection of social performance preservation may be associated with the work functioning of the role of the individuals in the family, at work and, consequently, in society, since the study involved patients who maintained their work activities during the proposed treatment protocol.

It should be noted that there was no significant differences in the WRFQ-Br scores of the individuals classified according to the treatment protocols. For the latter comparison, patients who underwent radiotherapy and hormone therapy were excluded, because they were a group made up of a small number of individuals.

In regard to the other scores, probably due to technological breakthroughs in the area and improved intervention techniques – drugs and surgery – the patients registered work functioning and capacities similar to those of the population in general.

A study conducted by Braithwaite *et al*. [27] showed that about 39% of women diagnosed and treated for breast cancer had some functional limitation due to inflammatory processes or reduced functions of organs and systems. The evaluation of these limitations may be an important factor in the clinical assessment and also in creating a better quality of life for these patients. It is also known that women with diagnosis and treatment of breast neoplasms present better physical work functioning than those diagnosed with pulmonary neoplasia. Other chronic comorbidities and depressive symptoms may directly affect physical work functioning [28].

The persistence of physical limitations in childhood cancer survivors is known, hindering work functioning in daily activities and sports. The reports are related to treatments involving especially radiotherapy, for bone, central nervous system, and retinoblastoma neoplasias [29].

It is important to emphasise that the time for partial or total return to work depends not only on the type of cancer but also on occupational factors related to each individual’s activities [30].

Patients, in general, seek to increase their survival, while health professionals aim to provide conditions to improve quality of life [31]. It is important and necessary to align the objectives of the multidisciplinary health team with the expectations of the patients undergoing oncological treatment.

The study was limited by the small number of individuals studied (*n* = 51), due to the large number of work-related absences, elderly people (on retirement), and deaths.

## Conclusion

The work functioning assessment of workers diagnosed with cancer undergoing radiotherapy using WRFQ-Br showed higher scores for social demands, probably related to the well-known social support received, and lower for physical demands, linked to functional limitations deriving from previous surgical interventions or due to the clinical profile itself.

In addition, the study and the services offered in the clinical practice of the researchers raised the hypothesis that, also in Brazil, patients undergoing cancer treatment have greater support from the multidisciplinary team in terms of health, family, occupation and government, which provides better physical, psychological, and social conditions for the leave of absence and for a return to work.

There is a need for studies to monitor these patients longitudinally from the initial diagnosis, enabling the assessment of work functioning before the leave from work, during treatment and in planning the return to work and adaptations required for the return.

It is hoped that this study will contribute towards understanding the importance of using validated tools for the global assessment of individuals with cancer, and in creating awareness regarding the availability of an instrument to assess workers in a way that enables them to maintain or return to their work activities, which has a significant and diverse role in patients’ daily activities and in the rest of their life.

## Confiict of interest

The authors declare no conflicts of interest.

## Figures and Tables

**Table 1.** Characterisation of workers submitted to radiotherapy (*n* = 51). Campinas, SP, 2013.

Variables	*n*(%)	Average	Dp*	Minimum	Median	Maximum
**Age (years)**		53.35	10.08	30.00	53.00	72.00
**Gender** Male Female	23 (45.10) 28 (54.90)					
**Education Level** Primary school Secondary school Higher education Postgraduate level	9 (17.65) 6 (11.76) 28 (54.90) 8 (15.69)					
**Work load**		41.45	10.04	20.00	40.00	65.00
**Time on the Job**		20.31	11.92	10.00	20.00	50.00
Type of work** Manual Mixed Not manual	13 (25.49) 21 (41.18) 17 (33.33)					
**Type of neoplasia**						
Breast	23 (45.10)					
Prostate	12 (23.53)					
Lymphoma, non-Hodgkin	3 (5.88)					
Rectum	2 (3.92)					
Uterus	2 (3.92)					
Parotid gland	2 (3.92)					
Stomach	2 (3.92)					
Pharynx/Hypopharynx	2 (3.92)					
Skin	1 (1.96)					
Spine/Bone	1 (1.96)					
Bladder	1 (1.96)					
**Treatment protocols**						
Exclusive radiotherapy	8 (15.69)					
Radiotherapy and chemotherapy	9 (17.65)					
Radiotherapy and hormone therapy	3 (5.88)					
Radiotherapy and surgery	14 (27.45)					
Radiotherapy, chemotherapy, and surgery	17 (33.33)					

* dp: desvio-padrão (standard deviation)

** Classification of work according to Hébert (1996).

**Table 2.** Average assessment work functioning values with the WRFQ-Br of worker’s undergoing radiotherapy. Campinas, SP, 2013 (*n* = 51).

Subscale	Average	dp*	Minimum	Median	Maximum
Work plan	91.51	15.45	30.00	100.00	100.00
Production demand	90.20	14.75	42.85	96.42	100.00
Physical demand	86.35	15.78	41.66	91.66	100.00
Mental demand	89.05	16.57	25.00	95.83	100.00
Social demand	94.33	11:47	41.66	100.00	100.00
Total score	87.95	16.70	10.00	92.73	100.00

* dp: desvio padrão (standard deviation)

**Table 3.** Average assessment work functioning values with the WRFQ-Br of worker’s undergoing radiotherapy. Campinas, SP, 2013 (*n* = 51).

Demand/treatment protocol	Rt^1^			Rt^1^ and Qt^2^			Rt^1^ and cir^3^			Rt^1^ Qt^2^ and Cir^3^			p value^5^
	*N*	Average	dp^4^	*N*	Average	dp^4^	*N*	Average	dp^4^	*N*	Average	dp^4^	
Work plan	8	91.88	9.61	9	95.80	6.36	14	82.86	25.7	17	95.59	8.46	0.3838
Production	8	93.69	9.88	9	89.68	16.51	14	84.82	22.05	17	93.48	7.72	0.9652
Physics	8	92.19	7.55	9	86.11	15.90	14	86.84	18.34	17	82.50	17.73	0.7442
Mental	8	86.04	15.50	9	92.13	14.20	14	85.42	25.27	17	91.37	9.26	0.6258
Social	8	96.87	6.20	9	94.44	11.03	14	91.07	17.74	17	96.87	6.72	0.9285
Total score	8	92.00	7.02	9	90.37	11.37	14	79.71	28.23	17	91.09	7.40	0.9361

1 Rt: radiotherapy;

2 Qt: chemotherapy (Quimioterapia);

3 Cir: surgery (Cirurgia);

4 Dp: standard deviation (Desvio padrão);

5 Kruskal–Wallis test.
